# Evolving Natural History of Metastatic Prostate Cancer

**DOI:** 10.7759/cureus.11484

**Published:** 2020-11-14

**Authors:** Nellie N Nafissi, Heidi E Kosiorek, Richard J Butterfield, Cassandra Moore, Thai Ho, Parminder Singh, Alan H Bryce

**Affiliations:** 1 Internal Medicine, Mayo Clinic, Scottsdale, USA; 2 Statistics, Mayo Clinic, Scottsdale, USA; 3 Hematology/Oncology, Mayo Clinic, Scottsdale, USA

**Keywords:** visceral, imaging, progression, surveillance, survival, metastatic

## Abstract

Introduction

The systemic therapies available to patients with metastatic prostate cancer (mPC) have improved dramatically over the past decade. Anecdotal experience suggests that the increased available lines of therapy have changed the profile of mPC to include a higher prevalence of visceral metastases.

Materials and Methods

A retrospective review of 472 patients with prostate cancer who died in 2009 and in 2016 was performed. Patients with metastatic disease who had imaging within six months of death were included. A total of 164 patients were eligible for analysis.

Results

Overall rates of visceral and distant metastases, including the lung, liver, adrenal, brain, renal, spleen, and thyroid, were higher in patients who died in 2016 as compared to those who died in 2009 (40.0% and 26.1%, respectively, p-value = 0.07). Forty-four percent of patients who died in 2016 used five or more lines of systemic treatments compared to 26.1% of patients in 2009.

Conclusion

The emergence of new systemic therapies for mPC is changing the natural history of the disease. Visceral metastases are being seen with increasing frequency than in the past. This observation is important for clinicians who are monitoring patients with prostate cancer to maintain a high suspicion for visceral disease.

## Introduction

Despite many recent advances in therapy, prostate cancer continues to be the second leading cause of cancer-related death for men in the United States [1]. From 2008 - 2013, the five-year relative survival rate for all stages combined was 99% [1]. However, for men with distant disease, the five-year relative survival rate was only 30%. One in nine men diagnosed with prostate cancer is expected to develop invasive disease. The most common site of metastasis for prostate cancer is bone, occurring in 80% to 90% of men with metastatic prostate cancer (mPC) [2-3]. Prior literature reported visceral disease to occur infrequently. A population-based analysis found that only 10% of men with mPC to have liver metastasis [2]. However, anecdotal experience suggests that visceral disease has become more prevalent.

The systemic therapies available to patients with mPC have improved dramatically over the past decade. Prior to 2010, the only agents with a proven survival benefit for patients with metastatic disease were androgen deprivation therapy, anti-androgens, and docetaxel. In April 2010, sipuleucel-T-T was Food and Drug Administration (FDA) approved for mPC following a phase III trial showing a 4.1-month improvement in median survival [4]. Shortly thereafter, cabazitaxel was approved in June 2010 after showing a 30% reduction in the risk of death and an improved median overall survival compared with mitoxantrone [5]. In 2011, abiraterone was FDA approved following studies showing a 35.4% reduction in the risk of death after one year [6]. Enzalutamide was FDA approved in 2012 following a phase III trial showing overall survival of 18.4 months in the enzalutamide group versus 13.6 months in the placebo group [7]. Lastly, radium-223 was FDA approved in 2013 after a phase III trial showed a median overall survival benefit of 3.6 months in men with mPC and bone metastases [8].

Anecdotal experience suggests that the increased available lines of therapy have changed the profile of mPC to include a higher prevalence of visceral metastases, in addition to bone and lymph node metastases. In this retrospective study, we report the sites of metastases in patients with mPC who died in 2009 compared to those who died in 2016 in order to quantify the changing prevalence of visceral disease. We hypothesize that the proliferation of life-prolonging treatments has changed the end-stage disease burden among patients with metastatic prostate cancer.

Of note, this abstract was previously presented at the American Society of Clinical Oncology Annual Meeting in June 2019 (http://meetinglibrary.asco.org/record/175854/abstract).

## Materials and methods

This retrospective study was completed with the approval of the Mayo Clinic Institutional Review Board (approval #17-008872). Data were extracted from the Mayo Clinic cancer registries across three sites (Rochester, Arizona, and Florida). We identified patients with metastatic prostate cancer who died in 2009 and in 2016.

Continuous variables were compared by Wilcoxon rank-sum test and categorical data by Fisher’s exact or Chi-square tests. The Statistical Analysis System (SAS) software, version 9.4 (SAS Institute Inc., Cary, NC, USA) was used for analysis. P-values < 0.05 were considered statistically significant.

## Results

**Figure 1:**
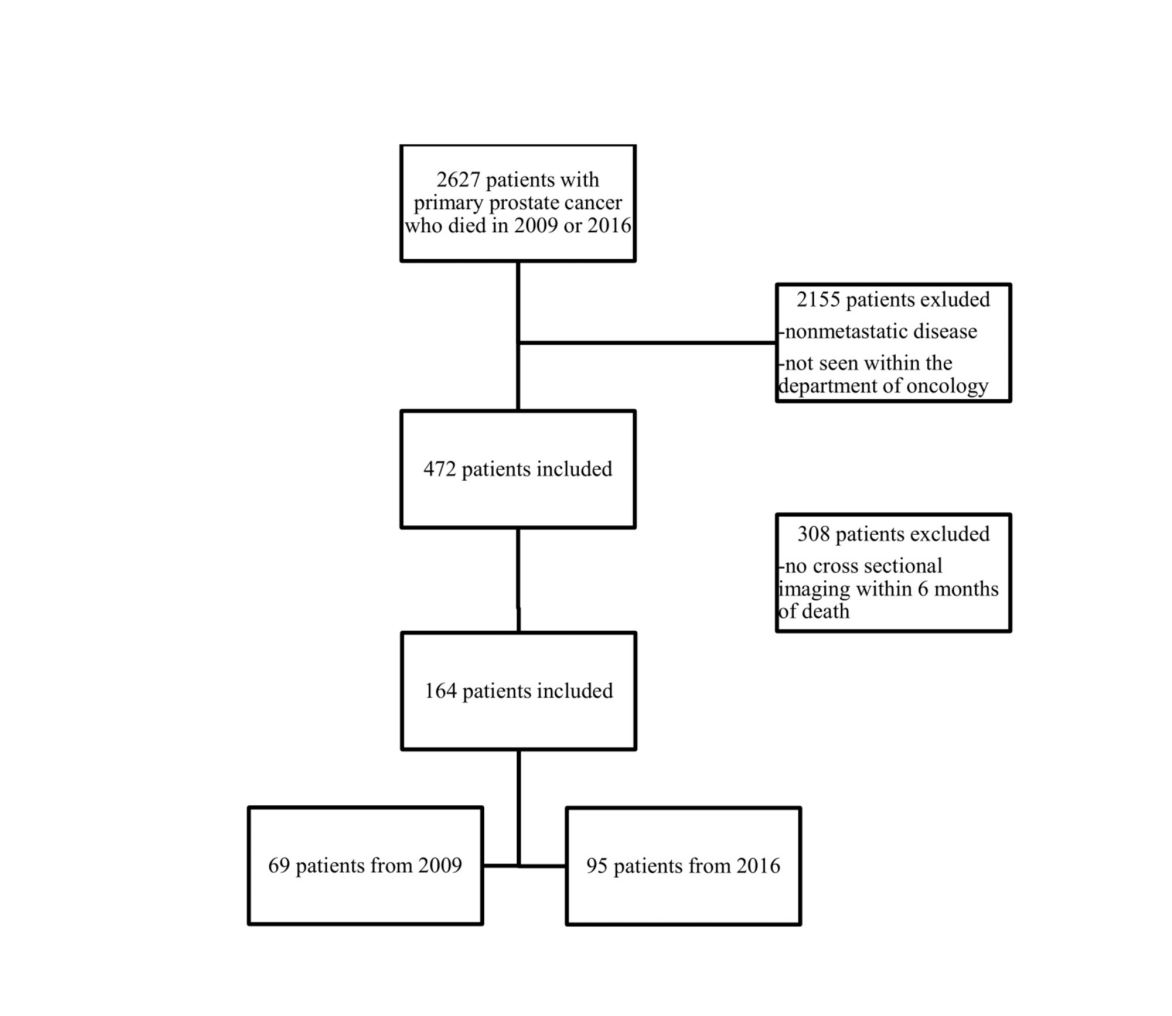
Patient inclusion and exclusion criteria

There were 2,627 patients with primary prostate cancer who expired in 2009 and 2016. Of those patients, 472 were seen in the Department of Oncology with metastatic disease, with 205 in the 2009 cohort and 267 in the 2016 cohort. After identifying patients who had imaging within six months of death, 164 patients were included in this study. Sixty-nine patients were from 2009 and 95 from 2016 (Figure 1). We chose six months as an arbitrary measure in an effort to ensure that imaging represented disease burden near the end of life.

**Figure 2:**
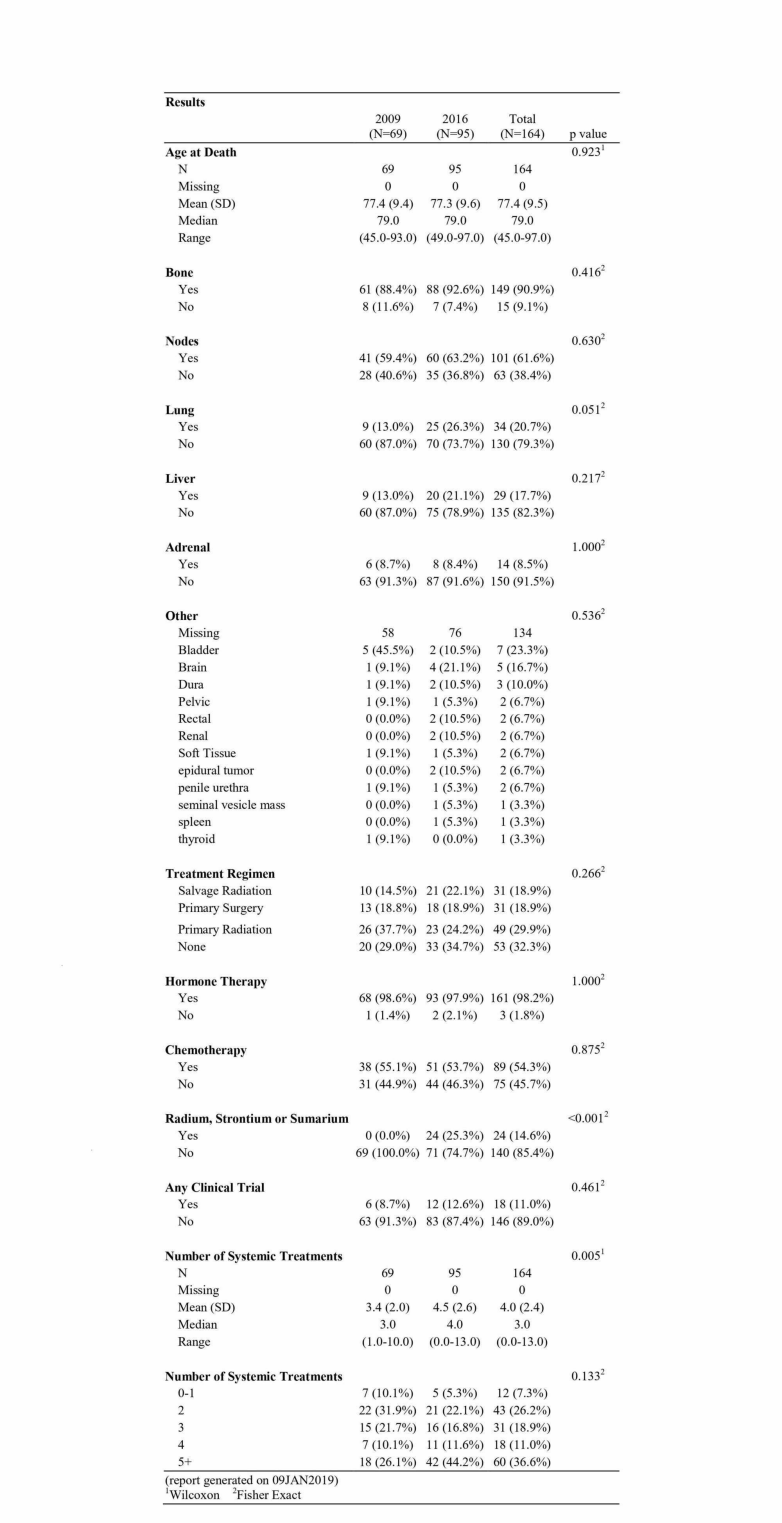
Locations of metastatic disease and treatments received ^1^Wilcoxon rank-sum test ^2^Fisher's exact text N: number; SD: standard deviation

The mean age at death overall was 77.4 years (standard deviation (SD): 9.5) and did not differ significantly by cohort. Bone metastases were present in 88.4% of patients in 2009 and in 92.6% of patients in 2016. Rates of nodal metastases were also similar, with 59.4% in 2009 and 63.2% in 2016. Lung metastases were present in 13.0% of patients in 2009 and in 26.3% of patients in 2016, reaching near statistical significance with p = 0.051. Liver metastases were also increased in 2016 at 21.1% compared to 13.0% in 2009. Adrenal gland metastases were relatively prevalent with 8.7% in 2009 and 8.4% in 2016. Other notable sites of metastases included brain, kidney, and spleen. There were four (21.1%) instances of brain metastases in 2016 compared to one (9.1%) in 2009. There were two patients with renal metastases in 2016 compared to none in 2009. One patient was noted to have a splenic metastasis in the 2016 cohort. Overall rates of visceral and distant metastases, including lung, liver, adrenal, brain, renal, spleen, and thyroid, were higher in patients who died in 2016 as compared to those who died in 2009 (40.0% and 26.1%, respectively, p = 0.07). Overall in both cohorts, 39 patients (23.8%) had metastatic disease at the time of the initial diagnosis. Of the patients who died in 2016, 31 had de novo metastatic disease compared to only eight in the 2009 cohort (Figure 2).

Patients who died in 2009 received a median of three (range: 1 - 10) systemic treatments versus four (range: 0 - 13) in those who died in 2016 (p = 0.005). Forty-four percent of patients who died in 2016 used five or more lines of systemic treatments compared to 26.1% of patients in 2009. There were similar rates of prostatectomy in patients in 2009 and in 2016 (18.8% and 18.9%, respectively). Notably, primary radiation was used in 37.7% of the patients in 2009, and in 24.2% of the patients in 2016. Nearly all patients received anti-androgen therapy in both 2009 and 2016, 98.6% and 97.9%, respectively.

## Discussion

This study shows the incidence of visceral metastases has significantly increased to 40% in 2016 from 26.1% in 2009. This is similar to other recent studies showing rates of visceral metastases from 40% in 2009 to 49% in 2016 [9-11]. Prior studies note visceral disease rates to be about 20% prior to 2010 [2, 12]. Patients also received more lines of therapy in 2016. Given the multiple systemic therapies with proven survival benefit approved since 2010, men are living longer with metastatic prostate cancer than they have in the past [13-14]. The increased lines of therapy may also be driving the increase in visceral disease that we observed in the past decade.

This changing natural history of metastatic prostate cancer is important for multiple reasons. First, visceral metastases are recognized as an ominous prognostic sign. Visceral disease, particularly liver metastases, are known to be a poor prognostic factor [12, 15-16]. A recent meta-analysis found the overall survival time for patients with bone metastases to be 21.3 months (95% CI, 20.8 to 21.9 months) as compared to 19.4 months for patients with lung metastases (95% CI, 17.8 to 20.7 months). The median overall survival time for patients with liver metastases was 13.5 months (95% CI, 12.7 to 14.4 months) [17].

This aggressive natural history has also led to the inclusion of visceral metastases as one of the seven eligibility criteria for the diagnosis of anaplastic carcinoma [18-19]. Other criteria include bulky lymphadenopathy, histologic evidence of small-cell prostate cancer, lytic bone metastases, and prostate-specific antigen (PSA) discordance. These patients have been found to respond well to platinum-based chemotherapy, in addition to docetaxel, as the treatment of choice.

Second, recognizing patterns of metastases should influence the pattern of surveillance. Routine surveillance of patients with prostate cancer in the community may include regular PSA testing and physical examination, with imaging performed only at times of PSA recurrence or new symptoms [11]. Even in patients with bone-only disease, soft tissue disease can develop during treatment. In patients treated with radium-223, up to 46% will develop soft tissue progression within three months of treatment [10, 20]. In the PREVAIL study (www.clinicaltrials.gov/ct2/show/NCT01212991), 40% of patients who started with bone-only disease upon initiation of enzalutamide developed soft tissue disease at the time of progression [11]. This study also demonstrated the phenomenon of PSA discordance which occurs in patients who have a progression of metastases without a concordant increase in PSA. A posthoc analysis of the PREVAIL study found an overall rate of radiographic progression without PSA progression of 24.5% [11]. However, in patients with lung or liver metastases, the rate of PSA discordance was found to be 34.4%. This data, in conjunction with the increasing prevalence of the visceral disease, reinforces the importance of screening with comprehensive imaging in addition to PSA, history, and physical exam. As of 2017, the Advanced Prostate Cancer Consensus Conference recommended routine imaging of patients with metastatic castrate-resistant prostate cancer with computed tomography (CT) scans and bone scintigraphy every three to six months [19]. However, this recommendation was supported by only 54% of the panel, indicating there is still debate on the standard of care.

Of note, a higher rate of de novo metastatic disease was present in the 2016 cohort than in 2009. In 2012, the United States Preventive Services Task Force (USPSTF) gave a grade D (discourage the use of this service) recommendation for prostate-specific antigen (PSA)-based prostate cancer screening for all age groups [21]. This large retrospective study of nearly 900,000 screen-eligible men from 2010 and 2015 found a 23.4% (95% CI 23.0 - 23.8%) reduction of screening rates between the two groups. It also noted that the rate of metastatic cancer at diagnosis had increased by 36.9% (95% CI 9.5 - 71%). This is consistent with our findings.

The limitations of our study include the retrospective nature of the data. The data is collected from three centers within one institution, which is largely a referral center. A selection bias may be present for patients with more aggressive disease being referred to the institution and thus more visceral metastases. However, our rate of visceral metastases of 40% is similar to findings from other studies, as reviewed in the section above. The sample numbers are relatively small, which makes it difficult for statistical significance to be reached for each site of metastases. There may also be a discrepancy in imaging techniques available in 2009 compared to 2016. This could lead to metastases found on imaging in 2016 which could have been missed in 2009 due to inferior imaging technology. However, we do not believe this is a contributing factor, as all patients from both cohorts underwent imaging with CT or magnetic resonance imaging (MRI). No advanced imaging modalities were included in the study.

Further areas for study would include differentiating if the observed increase in visceral disease is secondary to increased survival time or if it is an effect of certain classes of treatment selected for more aggressive disease.

## Conclusions

The emergence of new systemic therapies for mPC is changing the natural history of the disease. Metastatic disease was previously confined to the bone and regional lymph nodes with other rare sites of metastases. Forty percent of patients now develop visceral metastases compared to 26% in the past. This observation is important for clinicians as they must maintain a high suspicion of visceral metastatic disease, and surveillance of patients with prostate cancer should include routine imaging. Furthermore, this observation will drive the need for new treatment approaches targeting visceral metastases.
